# Comparison of risk-adjusted survival in two Scandinavian Level-I trauma centres

**DOI:** 10.1186/s13049-016-0257-9

**Published:** 2016-05-10

**Authors:** Poya Ghorbani, Kjetil Gorseth Ringdal, Morten Hestnes, Nils Oddvar Skaga, Torsten Eken, Anders Ekbom, Lovisa Strömmer

**Affiliations:** Division of Surgery, Department of Clinical Science, Intervention and Technology (CLINTEC), Karolinska Institute, Stockholm, Sweden; Department of Anaesthesiology, Vestfold Hospital Trust, Tønsberg, Norway; Norwegian Trauma Registry, Oslo University Hospital, Oslo, Norway; Oslo University Hospital Trauma Registry, Oslo University Hospital, Oslo, Norway; Department of Research and Development, Division of Emergencies and Critical Care, Oslo University Hospital – Ullevål, Oslo, Norway; Department of Anaesthesiology, Division of Emergencies and Critical Care, Oslo University Hospital – Ullevål, Oslo, Norway; Department of Medicine, Karolinska University Hospital – Solna, Stockholm, Sweden

**Keywords:** Trauma mortality, Scandinavia, Injury severity, TRISS, Risk-adjustment

## Abstract

**Background:**

Assessment of trauma-system performance is important for improving the care of injured patients. The aim of the study was to compare risk-adjusted survival in two Scandinavian Level-I trauma centres.

**Methods:**

This was an observational, retrospective study of prospectively-collected trauma registry data for patients >14 years from Karolinska University Hospital – Solna (KUH), Sweden, and Oslo University Hospital – Ullevål (OUH), Norway, from 2009-2011. Probability of survival (Ps) was calculated according to the Trauma and Injury Severity Score (TRISS) method. Risk-adjusted survival per patient was calculated by assigning every patient a value corresponding to gained or lost fractional life: Each survivor contributed a reward of 1-Ps and each death a penalty of -Ps. The sum of penalties and rewards, corresponding to the difference between expected and actual mortality, was compared between the centres. We present the data as excess survivors per 100 trauma patients.

**Results:**

There were 4485 admissions at KUH and 3591 at OUH. The proportion of severely injured patients was higher at OUH compared with KUH (Injury Severity Score [ISS] >15: 33.9 % vs. 21.1 %, *p* <0.001). OUH had a larger proportion of patients >65 years (16.0 % vs. 13.4 %, *p* <0.001) and greater comorbidity (ASA-PS ≥3: 14.6 % vs. 6.9 %, *p* <0.001) compared with KUH. The frequency of helicopter transport and presence of prehospital physicians was higher at OUH compared with KUH (27.6 % vs. 15.5 % and 30.5 % vs. 3.7 %, both *p* <0.001). Secondary admissions were 5.2-fold more common at OUH compared with KUH (*p* <0.001). There were no differences in 30-day mortality for severely injured patients (ISS >15). Risk-adjusted survival rate was higher at OUH than at KUH for primary (0.59 vs. 0.51) but lower for secondary (1.41 vs. 2.85) admissions (both *p* <0.001).

**Conclusion:**

Adjustments for age as a continuous variable and comorbidity should be made when comparing risk-adjusted survival between hospitals, but this is not possible with the TRISS model. A survival prediction model that takes this into account may be a better choice for Scandinavian trauma populations. The current study could not rule out the influence of the system differences between the centres on risk-adjusted survival.

## Background

Comparison of trauma-care performance between different national and/or international trauma systems is essential for understanding and improving a trauma system [1]. Trauma system and trauma centre performance are dependent upon trauma system structure and resources, geography, prehospital and hospital trauma care processes and patient case mix.

The incidence of major trauma is generally low in Scandinavia, and Scandinavian capitals share many similarities with regard to infrastructure, socio-politics and health care services [2–4]. The trauma care infrastructure is similar between Karolinska University Hospital - Solna (KUH) in Stockholm, Sweden, and Oslo University Hospital - Ullevål (OUH) in Oslo, Norway, and both centres are equivalent to a Level-1 Trauma Center [5]. Trauma registries are available in both centres, and trauma registry datasets are based upon the same European core dataset [6]. There are however few studies of epidemiological patterns, trauma care processes or outcomes, and no studies on comparison of trauma systems and trauma care between Scandinavian trauma centres.

The probability of survival (Ps) for a trauma patient is frequently estimated with the North American Trauma and Injury Severity Score (TRISS) method [7–9]. The method has been in worldwide use for more than 30 years. TRISS attempts to predict probability of patient survival based on the physiological status of the patient on hospital admission, overall anatomic injury severity, age and type of injury. There are however well known shortcomings of the model [10–12] and the most important limitation is the application of this prediction model to datasets other than the one from which the model was derived.

The primary aim of the current study was to compare TRISS risk-adjusted survival between KUH and OUH. The secondary aim was to compare patient-related factors and pre- and in-hospital trauma care processes of relevance for outcome comparison between the two trauma centres. Such a comparison could link together two Scandinavian trauma populations and registries, thus enabling the creation of a robust epidemiological foundation for future research in the Scandinavian trauma population.

## Methods

### Population

This was an observational, retrospective study of prospectively collected registry data at both centres from a three-year period from January 2009 through December 2011. During the study period, KUH had a catchment population of 1.9 million inhabitants from an area of 6,526 km^2^ [2, 13]. The trauma system in the Stockholm region consisted of seven acute-care hospitals, of which KUH was the sole major trauma centre. OUH was the major trauma centre in Oslo and the trauma referral centre for 2.7 million inhabitants in the South-Eastern Norway Regional Health Authority region, with an area of 111,000 km^2^. The regional trauma system consisted of 19 acute-care hospitals located outside Oslo [3, 4, 14]. The prehospital transport system that served KUH consisted of one helicopter emergency medical service (HEMS) base with one helicopter operational 24 h per day during the entire year and an additional helicopter available during daytime in the summer. Physicians were not available in the helicopters, but an anaesthesiologist-staffed ground ambulance was operational during daytime on weekdays. In the South-Eastern Norway Regional Health Authority region there were five HEMS bases with a total of six anaesthesiologist-staffed helicopters, all operating 24 h per day [15–17]. The HEMS bases also operated rapid-response cars. Additionally, there was an anaesthesiologist-manned rapid-response car operating in Oslo during daytime.

### Inclusion and exclusion criteria

All trauma patients >14 years admitted with trauma team activation, irrespective of Injury Severity Score (ISS) [18], and patients without trauma team activation with ISS >9 who were admitted to the hospital directly or transferred from a local hospital within 24 h after injury were included. Patients transferred to the trauma centres more than 24 h after injury were included only if the trauma team was activated upon patient arrival. Drowning, predominant burn injuries, and hypothermia without concomitant trauma were excluded. Data entry in the registries has been described previously [2–4]. The reporting of the study was designed to conform to the STROBE statement guidelines for reporting observational studies [19].

### Criteria for trauma team activation

Trauma team activation criteria were similar at both centres and based on specific anatomical injuries, mechanism of injury and physiologic derangement such as circulatory or respiratory instability or reduced level of consciousness, or other situations with a high index of concern. Patients with an isolated fracture of a single extremity were excluded unless the trauma team was activated.

### Data variables

#### Demography and injury severity

We collected data on age, sex, comorbidity classified as pre-injury American Society of Anesthesiologists Physical Status Classification System Score (ASA-PS) [20, 21], injury mechanism (blunt vs. penetrating), Abbreviated Injury Scale (AIS) [22, 23], ISS, New Injury Severity Score (NISS) [24] and the Revised Trauma Score (RTS) variables [25] on arrival.

#### Injury coding

In the Trauma Registry at OUH, all injuries were coded according to the AIS 2005-update 2008 (AIS08) [22]. At KUH, the injuries were classified according to the AIS 2005 (AIS05) version [23] through June 30, 2011, and according to AIS08 after that date.

#### Trauma care processes

Data regarding prehospital time (time from alarm until arrival at hospital), presence of a prehospital anaesthesiologist at scene of injury, prehospital intubation, type of transportation, emergency room intubations and CT scans, the first key life-saving emergency intervention performed, hospital and intensive care unit (ICU) length of stay (LOS), and whether the patient was a primary or secondary admission (transferred from a local hospital) was collected at both centres.

#### Mortality and risk-adjusted survival

30-day mortality and dead on arrival (DOA) were defined according to the Utstein Trauma Template and identified through clinical review [6, 26]. Probability of survival (Ps) was calculated according to the TRISS method using the coefficients from the 2009 revision [27]. Risk-adjusted survival per patient was calculated by assigning every patient a value corresponding to gained or lost fractional life, where survivors were given a value of 1 and those patients who died a value of 0. Each survivor thus contributed a reward of 1-Ps and each death a penalty of -Ps. The sum of penalties and rewards, corresponding to the difference between expected and actual mortality [3, 28], was compared between the centres. Data was presented as excess survivors per 100 trauma patients are equivalent to the W statistic [25]. Patients, who died in spite of a probability of survival ≥0.8, were considered to be unexpected deaths and would cause major penalties to the total risk-adjusted survival. Therefore, the subgroup of patients with Ps ≥0.8 was characterized and the distribution of Ps values between centres analysed.

### Ethical approval

At OUH, the Data Privacy Ombudsman for research deemed that the study was exempt from a requirement for informed consent because of the anonymity of the extracted data and the absence of any treatment study protocol. At KUH, the Regional Ethical Review Board in Stockholm approved the study.

### Statistical methods

Normally distributed data are presented as means with standard deviations (SD), and data that are not normally distributed are presented as medians with interquartile ranges (IQR). To facilitate comparisons with previous literature means with standard deviations were also calculated. Comparisons of continuous data were performed using the independent *t* test, the Mann–Whitney *U* test or the Wilcoxon/Kruskal Wallis test depending of the distribution of the data. Normality was tested using the Shapiro-Wilk test. Differences between categorical variables were evaluated with Fisher’s Exact Test. Statistical significance was assumed for two-sided *p* values <0.05. Data were analysed with SPSS (Statistical Package for the Social Sciences, Version 21.0.0, SPSS, Inc, Chicago, IL).

## Results

### Patient demography, injuries, and admissions

Descriptive statistics for the study populations are presented in Table 1. There were 4485 trauma admissions at KUH and 3591 at OUH, corresponding to 2.3 trauma patients per 1000 inhabitants in the KUH catchment region and 1.3 trauma patients per 1000 inhabitants in the OUH catchment region. Compared with KUH, OUH had a greater proportion of trauma patients over the age of 65 years (*p* <0.01) and a greater proportion of patients with higher levels of comorbidity (pre-injury ASA-PS ≥3) (*p* <0.001).

**Table 1:** Patient demography, injuries, admission and mortality

	KUH *n* = 4485	OUH *n* = 3591	*p*	U/M KUH	U/M OUH
Age (years)	39 (25–55)	40 (26–57)	<0.05		4
Age >65 years	603 (13.4 %)	576 (16.0 %)	<0.01		4
Pre-injury ASA-PS	1 (1–1)	1 (1–2)	<0.001	15	6
Pre-injury ASA-PS ≥3	308 (6.9 %)	522 (14.6 %)	<0.001	15	6
Pre-injury ASA-PS ≥3 for age >65 years	204 (34.1 %)	294 (51.3 %)	<0.001	4	3
Male	3113 (69.4 %)	2618 (72.9 %)	<0.001		
Blunt trauma	4139 (92.3 %)	3202 (89.2 %)	<0.001		
ISS	5 (1–13)	10 (4–18)	<0.001		
NISS	6 (3–17)	12 (4–27)	<0.001	71	
Injury mechanism					30
Transport accidents	1977 (44.1 %)	1474 (41.1 %)	<0.01		
Fall	1671 (37.2 %)	1124 (31.6 %)	<0.001		
Other	837 (18.7 %)	963 (27.1 %)	<0.001		
Injury intention				31	33
Self-inflicted	223 (5.0 %)	138 (3.9 %)	<0.05		
Assault	563 (12.6 %)	481 (13.5 %)	0.256		
Secondary admission	290 (6.5 %)	1236 (34.4 %)	<0.001	9	
Crude mortality	143 (3.2 %)	202 (5.6 %)	<0.001		
DOA	39 (21.4 %)	26 (11.4 %)	<0.01		

Numbers are median and interquartile range, or number and proportion (%)

*KUH* Karolinska University Hospital-Solna, *OUH* Oslo University Hospital-Ullevål, *U/M* Unknown/Missing, *ASA-PS* American Society of Anesthesiologists Physical Status Classification System, *ISS* Injury Severity Score, *NISS* New Injury Severity Score, *DOA* Dead on Arrival, as fraction of total deaths

**Fig. 1:**
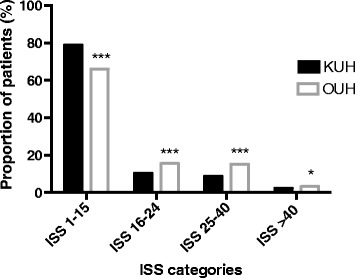
Proportions of patients in different ISS categories at KUH and OUH. ISS: Injury Severity Score; KUH: Karolinska University Hospital-Solna; OUH: Oslo University Hospital-Ullevål. *p <0.05, ***p <0.001 vs. KUH

The proportions of patients in the ISS subgroups 1–15, 16–24, 25–40 and >40 are presented in Fig. 1. Compared with KUH, OUH had a lower proportion of patients with minimal injuries (ISS 1) (16.6 % [n =596] at OUH vs. 25.6 % [n =1147] at KUH, *p* <0.001) and minor injuries (ISS <9) (41.8 % [n =1500] at OUH vs. 57.9 % [n =2598] at KUH, *p* <0.001).

Comparisons between primary and secondary admissions are presented in Table 2. Secondary admissions were 5.2 times more common at OUH compared with KUH (34.4 % vs. 6.5 %). At both KUH and OUH, the median age of secondary admitted patients was approximately ten years higher than in primary admitted patients. The secondary admissions also had higher ISS and NISS scores and slightly higher pre-injury ASA-PS score at both institutions (*p* <0.001).

**Table 2:** Comparison of age, comorbidity and injury severity in primary and secondary admissions at KUH and OUH

	KUH			OUH		
	Primary *n* = 4185	Secondary *n* = 290	*p*	Primary *n* = 2355	Secondary *n* = 1236	*p*
Age, years	38(25–54)	48(29–61)	<0.001	37(25–53)	46(27–64)	<0.001
ASA-PS	1(1–1)	1(1–2)	<0.001	1(1–2)	1(1–2)^a^	<0.001
ISS	5(1–11)	17(12–26)	<0.001	8(2–17)	14(8–22)	<0.001
NISS	6 (2–17)	27 (17–34)	<0.001	9 (2–22)	17 (10–29)	<0.001

Numbers are median and interquartile range

*KUH* Karolinska University Hospital-Solna, *OUH* Oslo University Hospital-Ullevål, *ASA-PS* American Society of Anesthesiologists Physical Status Classification System, *ISS* Injury Severity Score, *NISS* New Injury Severity Score

^a^Higher ASA-PS in secondary admissions at OUH

### Trauma care processes

Table 3 shows the data for pre- and in-hospital trauma care processes. The percentage of cases in which a prehospital anaesthesiologist was dispatched to the scene of the injury was 8.2 times higher for OUH patients than for KUH patients, and the prehospital intubation rate was 2.8 times higher for OUH patients. The higher prehospital intubation rate for OUH patients was only observed in primary admitted patients who were transported with helicopter (33.7 % [n = 227] vs. 7.7 % [n =53], *p* <0.001). The prehospital time for all primary admissions and for ground ambulance transport of primary admissions was shorter for OUH patients. In contrast, KUH had shorter helicopter transport times for primary admissions. The prehospital time variable however had a substantial number of missing values.

**Table 3:** Comparison of trauma care processes at KUH and OUH

	KUH (*n* = 4485)	OUH (*n* = 3591)	*p*	U/M KUH	U/M OUH
Prehospital time (min)^a^				123	300
Primary admissions	46 (37–58)	37 (24–57)	<0.001		
Ground ambulance missions	45 (36–57)	33 (22–52)	<0.001		
Helicopter ambulance missions	52 (43–62)	65 (48–90)	<0.001		
Prehospital transportations					23
Ground ambulance missions	3393 (75.7 %)	2552 (71.5 %)	<0.001		
Helicopter ambulance missions	697 (15.5 %)	984 (27.6 %)	<0.001		
Other	395 (8.8 %)	32 (0.8 %)	<0.001		
Prehospital anaesthesiologist at scene of injury	149 (3.7 %)	1088 (30.5 %)	<0.001	452	27
Prehospital intubations	126 (2.8 %)	280 (7.8 %)	<0.001		3
Emergency room intubations^b^	297 (6.8 %)	362 (10.9 %)	<0.001		
CT scans	4029 (89.8 %)	2901 (80.8 %)	<0.001		
CT for primary admissions	3788 (90.5 %)	2068 (87.8 %)	<0.01		
Key emergency interventions	327 (7.3 %)	326 (9.1 %)	<0.01		
ICU admissions	844 (18.8 %)	986 (27.5 %)	<0.001		
ICU LOS^c^ (days)	3 (1–7)	3 (2–10)	<0.01		
Hospital LOS (days)	1 (1–6)	3 (2–7)	<0.001	27	

Numbers are median and interquartile range, or number and proportion (%)

*KUH* Karolinska University Hospital-Solna; *OUH* Oslo University Hospital-Ullevål, *U/M* Unknown/Missing, *Prehospital time* Time from alarm (prehospital) to arrival to hospital, *LOS* Length of stay

^a^KUH; *n* = 4185, OUH; *n* = 2355

^b^KUH; *n* = 4359, OUH; *n* = 3308

^c^KUH; *n* = 844, OUH; *n* = 986

**Fig. 2:**
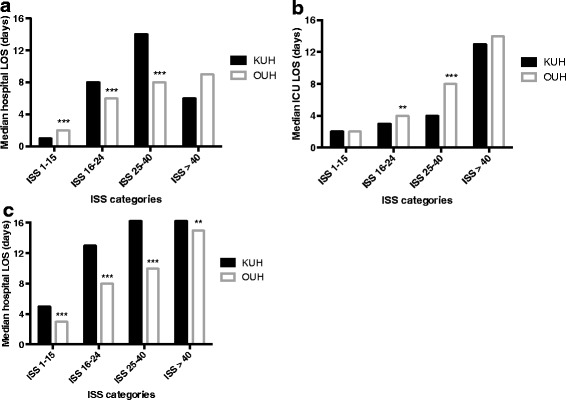
Median hospital LOS (**a**), median days in ICU (**b**) and median hospital LOS in patients that were admitted to ICU (**c**), at KUH and OUH stratified by ISS category. LOS: Length of stay; ICU: Intensive care unit; KUH: Karolinska University Hospital-Solna; OUH: Oslo University Hospital-Ullevål; ISS: Injury Severity Score. **p <0.01, ***p <0.001 vs. KUH

Trauma patients were more frequently admitted to the ICU at OUH than at KUH and median ICU and hospital LOS were longer (Table 3). Patients with less severe injuries (ISS 1-15) were admitted to the ICU more frequently at OUH than at KUH (17.0 % [n =403] vs. 8.3 % [n =294], *p* <0.001). The opposite was the case in the group with ISS 16-40 (45.9 % [n =506] vs. 57.8 % [n =488], *p* <0.001). There was no significant difference in ICU admissions in the group with ISS >40 between the two hospitals. Severely injured patients (ISS 16-40) had a longer median ICU LOS and a shorter median hospital LOS at OUH than at KUH (Fig. 2). For patients treated in the ICU, the median hospital LOS was shorter in all ISS categories at OUH compared to KUH.

### Mortality and risk-adjusted survival

**Fig. 3:**
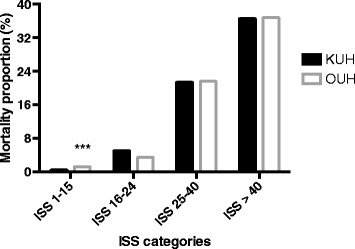
Mortality proportions for ISS categories at KUH and OUH. DOA patients are not included in this analysis. KUH: Karolinska University Hospital-Solna; OUH: Oslo University Hospital-Ullevål; ISS: Injury Severity Score; DOA: Dead on arrival. ***p <0.001 vs. KUH

The fraction of DOA patients was lower at OUH than at KUH, and the overall crude mortality rate was higher at OUH (Table 1). DOA patients were excluded in survival analyses. Except for the group of less severely injured patients (ISS 1–15), there were no differences in mortality in the different subgroups of injury severity (Fig. 3). TRISS risk-adjusted survival is shown in Table 4. For primary admissions and the total populations, median risk-adjusted survival was higher at OUH but mean risk-adjusted survival was higher at KUH. In contrast, both median and mean risk-adjusted survival was higher at KUH for secondary admissions. The differences were interpreted to be caused by different distributions of risk-adjusted survival between the two centres. Patients with Ps ≥0.8 who died (Table 5) caused major penalties to total risk-adjusted survival and the proportion of patients in this subgroup was larger at OUH than at KUH. The subgroup was characterized by a high number of secondary admissions with high median age and high comorbidity.

**Table 4:** Trauma and Injury Severity Score (TRISS) risk-adjusted survival at KUH and OUH

A	KUH	OUH	*p*
All patients	0.55 (0.32–1.88)	0.82 (0.40–2.94)	<0.001
Primary admissions	0.51 (0.32–1.58)	0.59 (0.34–2.07)	<0.001
Secondary admissions	2.85 (1.00–9.17)	1.41(0.55–3.99)	<0.001
B			
All patients	0.64 (14.8)	0.01 (19.7)	<0.001
Primary admissions	0.36 (1.4)	0.25 (1.8)	<0.001
Secondary admissions	4.49 (2.24)	−0.44 (0.63)	<0.001

Data are presented as excess survivors per 100 trauma patients compared to TRISS model predictions. A: median and interquartile range (IQR); B: mean and standard deviation (SD)

*KUH* Karolinska University Hospital-Solna, *OUH* Oslo University Hospital-Ullevål

**Table 5:** Characteristics of patients with probability of survival (Ps) ≥0.8 who died

	KUH	OUH	*p*
Number of patients (proportion of total population)	54 (1.2 %)	80 (2.2 %)	<0.001
Secondary admissions	7 (13 %)	42 (52.5 %)	<0.001
Age (years)	83 (69–90)	72 (46–83)	<0.01
Pre-injury ASA-PS ≥3	21 (39.6 %)	48 (60.8 %)	<0.05
ISS	21 (16–26)	25 (14–16)	0.400
NISS	27 (22–38)	34 (20–50)	0.148

Numbers are median and interquartile range, or number and proportion (%)

*KUH* Karolinska University Hospital-Solna, *OUH* Oslo University Hospital-Ullevål, *ASA-PS* American Society of Anesthesiologists Physical Status Classification System, *ISS* Injury Severity Score, *NISS* New Injury Severity Score

## Discussion

In this study, we found a survival benefit for OUH when comparing medians and for KUH when comparing means of risk-adjusted survival in the total trauma population and in primary admissions. In contrast, in secondary admissions, OUH had lower survival rate than KUH in both mean and median risk-adjusted survival. To summarize the demographic findings we observed that the trauma patients at OUH were 1) older with a higher comorbidity, 2) more severely injured, 3) more often transported from a local hospital to the trauma centre, and 4) more often transported with helicopter with a prehospital anaesthesiologist present compared to the trauma patients at KUH.

The differences between median and mean risk-adjusted survival in the total population in the present study can be explained by different case mix. It indicates a skewed distribution of probability of survival estimates. We explored this difference further in all patients at both trauma centres who died in spite of a probability of survival ≥0.8, thus contributing markedly to lower mean institutional survival rates. The patients in this group were of high median age and with high proportion of comorbidity. There was no difference in ISS between the two hospitals for this subgroup, but the OUH population had higher age, more comorbidity and a higher proportion of secondary admissions. There were 1.9 times as many patients in this group at OUH than at KUH, which contributed to the much lower mean of risk-adjusted survival at OUH. Age is a categorical variable in the TRISS model, with identical survival penalty for all patients older than 54 years. Comorbid patients have an increased mortality risk [21, 29] but comorbidity is not part of the TRISS model and therefore the probability of survival is overestimated in the comorbid trauma patient. A novel survival prediction model that includes age as a continuous variable and includes an adjustment for comorbidity has recently been validated [30, 31]. This model might be a better choice for use in Scandinavian trauma populations.

Secondary admissions may need to be analysed separately when comparisons of outcome are made, because the first treatment is given outside the trauma centre, and this will add to the heterogeneity within this subgroup compared to primary admissions. The median risk-adjusted survival was twice as high, and the mean risk-adjusted survival was 4.5 times higher for secondary admissions at KUH than at OUH. These differences were much larger than the differences observed in primary admissions. Secondary admissions at both hospitals were characterized by high age and higher level of comorbidity. The survival disadvantage for secondary admitted patients at OUH could be explained by the fact that there were more patients with a high TRISS probability of survival (Ps ≥0.8) transferred from other hospitals that died at OUH. The unsatisfactory adjustment for age (<55 and ≥55 years) in the TRISS model is suboptimal, especially for analyses in the secondary admission group with a median age of 83 and 72 years (KUH and OUH respectively).

Taken together, our data indicate that the observed differences in survival between the trauma centres could be a result of unsatisfactory adjustments for age and comorbidity, but it is not possible to rule out the influence of the system differences between the centres on mortality.

In the current study, we did not investigate the relationships between predicted survival and trauma care processes, but some of the differences in care processes observed may be of relevance for outcome. The percentage of prehospital physicians at the scene of injury was higher at OUH. For OUH patients, an anaesthesiologist was present at scene for every third trauma patient. This may be one reason for the higher intubation rate in the patients admitted directly to the trauma centre. It is also possible that medical resuscitation initiated by an anaesthesiologist during transportation to hospital, influenced physiology (i.e. RTS) on arrival and consequently biased the TRISS-based risk-adjusted survival in these patients in the current study.

The higher admission rate to ICU and the longer ICU LOS for severely injured patients (ISS 16–40) at OUH may be of relevance when comparing outcomes between KUH and OUH. However, ICU admission and ICU LOS are care processes that are not only influenced by injury severity, but also by treatment guidelines, resources (*e.g.* the availability of ICU beds) and discharge destination (*e.g.* transfer back to local hospital after stabilization). Therefore, both ICU admission rate and ICU LOS are difficult to relate to outcome.

There are also several important differences in the organization of the prehospital transport and medical care system between Norway and Sweden. In Norway, a national government-funded air ambulance system [32] provides rapid access to advanced life support by specially trained prehospital anaesthesiologists [15]. The vast geographical area covered by OUH (17 times larger than that of KUH) may be one of the reasons for the greater use of helicopters, longer prehospital time, higher percentage of secondary admissions and the higher percentage of prehospital physician attendance at OUH compared to KUH.

Two major differences between KUH and OUH were the markedly higher frequency of severely injured patients at OUH and the large number of minimal and minor injuries at KUH. There are several possible explanations for these observed differences. First, the catchment population for OUH was 1.4 times larger and the number of referral hospitals was higher compared to KUH (19 vs. six hospitals). This implies a greater possibility to better select and direct a larger number of severely injured trauma patients to OUH, whereas the less injured patients were treated in the local hospitals. The five-fold higher frequency of secondary admissions at OUH compared to KUH, with more severe injuries compared to primary admissions, reflects these regional differences. Second, the higher median ISS among the primary admissions at OUH compared to KUH could imply that prehospital triage was more accurate in directing severely injured patients to the trauma centre in Oslo compared to Stockholm. The higher presence of prehospital anaesthesiologists at scene of injury (30.5 % of all transports at OUH) found in the present study may have contributed to a more correct prehospital triage.

The current study has both strengths and limitations. A retrospective design may affect and potentially reduce the quality of the data. However, all trauma registry data were acquired prospectively, the trauma registries are based upon the same core dataset, and the amount of missing data was small. Without an inter-rater reliability test prior to data comparison, we cannot rule out some minor differences in coding practice between the two trauma registries, but the Utstein Trauma Template used by both registries is meant to minimise such differences. The anatomic injury classification differed (AIS05 vs. AIS08) during the first part of the study period. It has been shown that different AIS versions (*e.g.* AIS98 vs. AIS08) are not always comparable [33] but similar comparisons between AIS05 and AIS08 have not been made. Thus, we cannot rule out that the differences in anatomic injury classification may have disturbed the comparison.

## Conclusion

Adjustments for age as a continuous variable and comorbidity should be made when comparing risk-adjusted survival between hospitals, but this is not possible with the TRISS model. A survival prediction model that takes this into account may be a better choice for Scandinavian trauma populations. The current study could not rule out the influence of the system differences between the centres on risk-adjusted survival.

## Abbreviations

AIS: Abbreviated Injury Scale
ASA-PS: American Society of Anesthesiologists Physical Status Classification System Score
DOA: Dead on arrival
HEMS: Helicopter emergency medical service
ICU: Intensive care unit
IQR: Interquartile range
ISS: Injury Severity Score
KUH: Karolinska University Hospital – Solna
LOS: Length of stay
NISS: New Injury Severity Score
OUH: Oslo University Hospital – Ullevål
Ps: Probability of survival
RTS: Revised Trauma Score
TRISS: Trauma and Injury Severity Score
